# Alzheimer’s disease, mild cognitive impairment, and normal aging distinguished by multi-modal parcellation and machine learning

**DOI:** 10.1038/s41598-020-62378-0

**Published:** 2020-03-25

**Authors:** Jinhua Sheng, Meiling Shao, Qiao Zhang, Rougang Zhou, Luyun Wang, Yu Xin

**Affiliations:** 1 College of Computer Science, Hangzhou Dianzi University, Hangzhou, Zhejiang 310018 China; 2 Key Laboratory of Intelligent Image Analysis for Sensory and Cognitive Health, Ministry of Industry and Information Technology of China, Hangzhou, Zhejiang 310018 China; 3 Beijing Hospital, Beijing, 100730 China; 4 College of Mechanical Engineering, Hangzhou Dianzi University, Hangzhou, Zhejiang 310018 China; 5 Mstar Technologies Inc., Hangzhou, Zhejiang 310018 China

**Keywords:** Alzheimer's disease, Experimental models of disease

## Abstract

A 360-area surface-based cortical parcellation is extended to study mild cognitive impairment (MCI) and Alzheimer’s disease (AD) from healthy control (HC) using the joint human connectome project multi-modal parcellation (JHCPMMP) proposed by us. We propose a novel classification method named as JMMP-LRR to accurately identify different stages toward AD by integrating the JHCPMMP with the logistic regression-recursive feature elimination (LR-RFE). In three-group classification, the average accuracy is 89.0% for HC, MCI, and AD compared to previous studies using other cortical separation with the best classification accuracy of 81.5%. By counting the number of brain regions whose feature is in the feature subset selected with JMMP-LRR, we find that five brain areas often appear in the selected features. The five core brain areas are Fusiform Face Complex (L-FFC), Area 10d (L-10d), Orbital Frontal Complex (R-OFC), Perirhinal Ectorhinal (L-PeEc) and Area TG dorsal (L-TGd, R-TGd). The features corresponding to the five core brain areas are used to form a new feature subset for three classifications with the average accuracy of 80.0%. Results demonstrate the importance of the five core brain regions in identifying different stages toward AD. Experiment results show that the proposed method has better accuracy for the classification of HC, MCI, AD, and it also proves that the division of brain regions using JHCPMMP is more scientific and effective than other methods.

## Introduction

Alzheimer’s disease (AD) is the most common type of neurodegenerative disorder characterized by progressive impairment of memory and other cognitive functions in elderly people worldwide, and results in elderly people to death eventually. Pre-clinical stage of Alzheimer’s disease, also known as mild cognitive impairment (MCI), is a transitional state between normal aging and AD, often an early warning signal of AD. The correct recognition of MCI and AD plays an important role in the prevention, early detection and intervention of AD, and lays a foundation for the exploration of effective treatment methods for AD in the future.

The Human Connectome Program (HCP) proposed a multi-modal parcellation (MMP)^1^ of the human cerebral cortex with 180 areas per hemisphere. The HCPMMP is based on surface-registered multi-modal MR acquisition and objective semi-autonomic nerve anatomy, and the criteria are sharp changes in cortical architecture, function and connectivity. A range of studies^2–4^ have demonstrated that the widespread application of HCPMMP can help to understand the healthy brain and dementia, such as AD, schizophrenia (SCZ), Parkinson’s disease (PD). Some studies have shown that the brain connectivity for neurodegenerative diseases has changed significantly, and the topological structure of the brain network has been disrupted^4–8^. Network measurement of different regions of the human brain is considered to be an effective feature for recognition of cognitive impairment patients^9^. The HCPMMP sample was derived from 210 healthy adults but there are few studies on the cerebral cortex of AD patients. JHCPMMP introduced HCPMMP into the cerebral cortex of AD patients and applied it to the classification of HC, MCI, and AD^10^. The main goal of our study is to demonstrate an automated and accurate method for identification of AD, MCI and HC.

## Method

**Figure 1:**
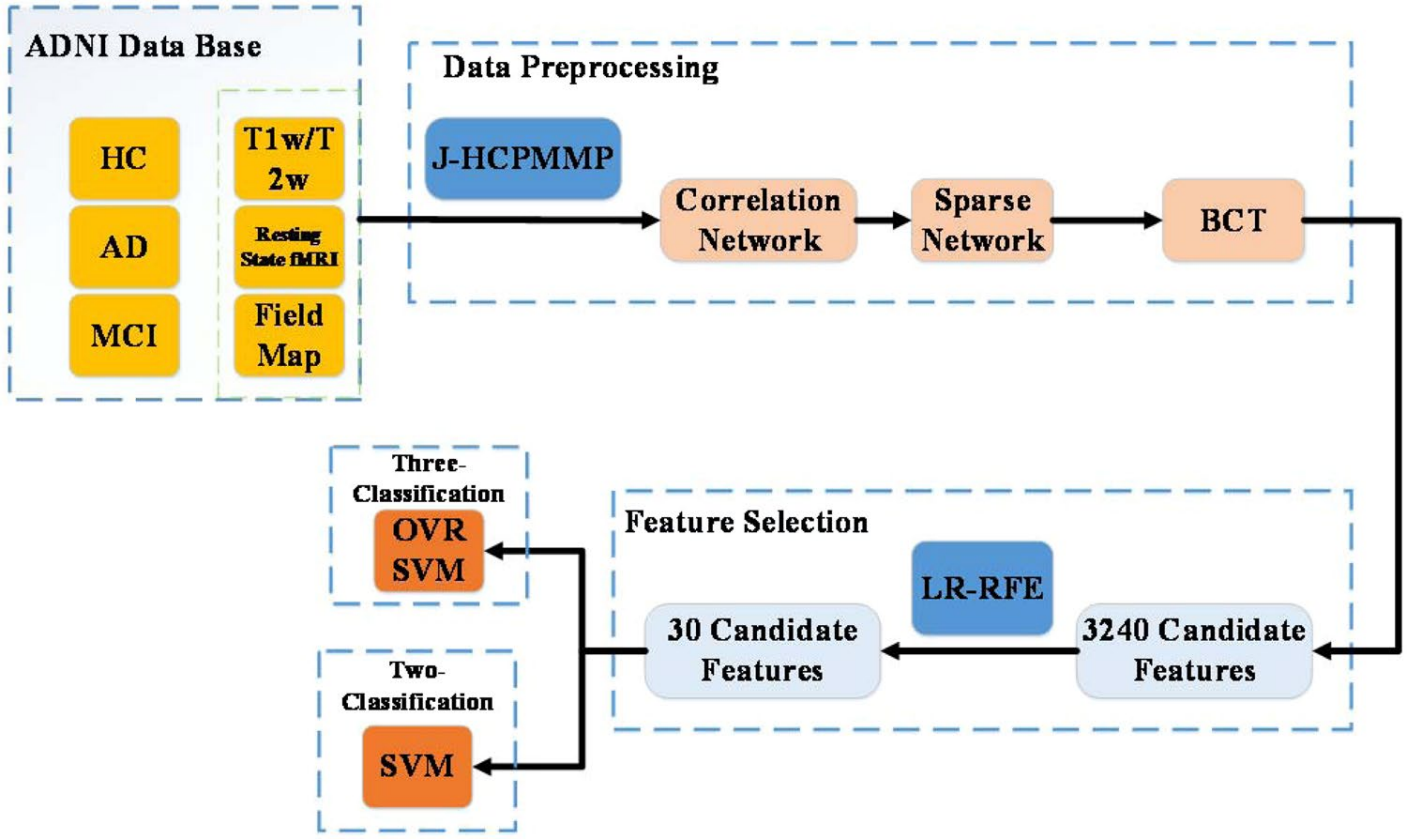
The process of the three-class classification.

A novel classification approach is proposed to accurately identify different stages toward AD by integrating the JHCPMMP with the logistic regression-recursive feature elimination (LR-RFE), which is named as JMMP-LRR. This method is applied to complete the entire experiment. Firstly, the sparse network is obtained by using JHCPMMP^10^. The process of this step is to process the fMRI data, project it to CIFTI Space, and obtain the sparse network through MMP. Secondly, we calculate the 9 attributes of brain networks, including strength, betweenness centrality, local efficiency etc, and obtain 3,240 candidate features of each subject. Subsequently, we apply LR-RFE to select the 30 features of each subject. Finally, the classifier of OVR-SVM is applied to classify the extracted features of HC, MCI and AD for classification. The process of the three-class classification in this paper is shown in Fig. 1.

### Data preprocessing

The brain is parcellated with 180 areas per hemisphere by using HCPMMP atlas, which delineates the cortical architecture, function, and connectivity. The sparse network is obtained with the help of JHCPMMP^10^. The process of this step is to process the fMRI data, project it to CIFTI Space, and obtain the sparse network through MMP. MMP can show dramatic changes in cortical thickness, myelin atlas, task fMRI, and resting fMRI for each brain region. The correlation can be calculated for 360 areas. The sparse network is generated by searching the proportion of the strongest weights (PSW). The purpose of this step is to reduce noise and weakly correlated connections.

Network features in each node of the connectivity network are calculated as the candidate features. The feature vector of each sample contained strength (S), betweenness centrality (BC), clustering coefficient (CC), local efficiency (LE), eigenvector centrality (EC), k-coreness centrality (KC), page rank centrality (PC), Subgraph centrality (SC) and flow coefficient (FC). The software calculating graph theoretical measures can be the Brain Connectivity Toolbox (BCT, available at: https://sites.google.com/site/bctnet/).

For single local network measure, a vector of 360 × 1 is formed in which each vector represents an eigenvalue from the corresponding functional area in brain cortex. By calculating the attributes of the brain network, a feature matrix of 360 × 9 is formed, and each feature is stored in a column. The advantage of scaling each column of the eigenvalue matrix is that the range of the eigenvalue is not too large, which leads to the dominance of the more valuable features in classification. Each feature is normalized to the range^−1^ ^1^. Therefore, the 360 × 9 = 3,240 candidate features are generated for classification of HC, MCI and AD.

### Feature selection

The network-based measure generates the 3,240 candidate feature values for classification, which greatly affects the calculation cost and classification accuracy. Then, the 3,240 candidate features was a feature vector for each subject. Noisy and irrelevant features often lead to over-fitting problem. Generally, feature selection should be implemented before classification by extracting a subset of feature from the original 3,240 candidate features, which could reduce training time, test time and improve classification performance.

There are two main methods for feature selection, including filter, wrapper. The characteristic of filter feature selection is to select features from data first, and then train the learner, the process of feature selection is independent of subsequent learners. The wrapper method uses an inductive algorithm directly to evaluate the feature subset, which is generally better than filter method in terms of prediction accuracy, but usually more computationally intensive.

Recursive feature elimination (RFE) is a common method in wrapper feature selection. From the final performance of the learner, the wrapped feature selection is better than the filtered feature selection. The RFE method continuously eliminates the features with low contribution scores on the basis of the iterative method, and then ranks each feature in each cycle to delete the n features with the lowest score.

**Figure:**
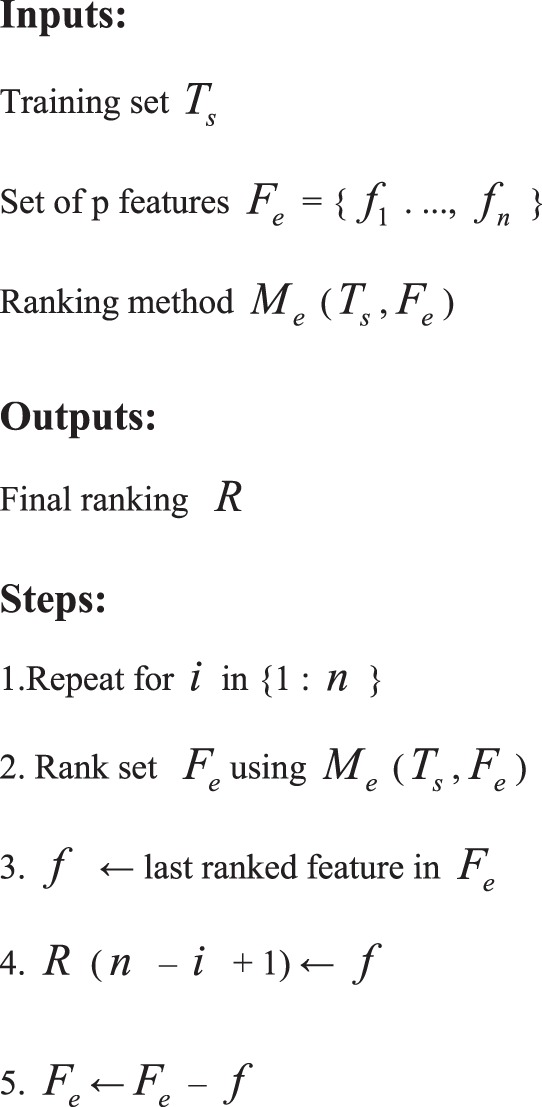
**Pseudo-code for the Recursive Feature Elimination (RFE) algorithm.**

LR-RFE algorithm is applied to extract important features from the 3,240 features. The main idea of LR-RFE algorithm is to repeatedly eliminate features with low contribution scores based on the iterative method, and rank each feature in each cycle using LR algorithm to delete the 10 features with the lowest score. The process is repeated for the remaining features until all features are traversed. From the 3,240 features, 30 optimal feature subsets are selected by using LR-RFE. The LR-RFE algorithm is implemented for finding optimal feature subset in Python using the Sklearn package.

**Figure:**
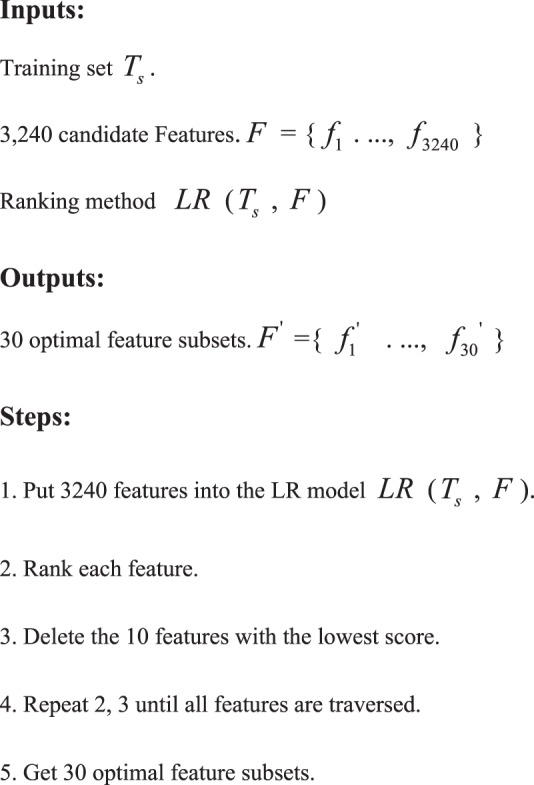
**LR-RFE algorithm steps.**

### SVM classifier

One-vs-the-rest support vector machine (OVR-SVM) is applied to achieve high classification accuracy after the dimension of the features has been reduced by LR-REF. SVM is a binary classification model to find a hyperplane to segment the samples. Dealing with multi-class classification problems requires the construction of a suitable multi-class classifier. This paper adopts OvR multi-class strategy, also known as one-vs-all.

OvR is the most commonly used strategy for multi-class classification. One class at a time is taken as a positive example, and the other classes are taken as a negative example to train N classifiers. If only one classifier is predicted as a positive class, the corresponding class label is used as the final classification result. The OvR-SVM is a multivariate statistical method that can be used for classification. In this paper, we use OVR-SVM as the classifier.

The mathematical principle of OVR-SVM is as follows: When you want to distinguish K classes, the problem can be expressed as the mathematical problem described in Eqs. 1–3^11^.

1 $$\begin{array}{c}{\min }_{{w}^{ij},{b}^{ij},{\xi }^{ij}}\,\frac{1}{2}{({w}^{ij})}^{T}{w}^{ij}+C\sum _{t}{\xi }_{t}^{ij}{({w}^{ij})}^{T}\\ \,\,\,\,\,\,\,\,\,\,\,\,\,\,\,\,\,\,\,\,\,\,\,\,\,\,\,\,\,\,\,\,\,\,\,\,\,\,\,\,\,\,\,\,\,\,\,\,\,\,{({w}^{ij})}^{T}\phi ({x}_{t})+{b}^{ij}\ge 1-{\xi }_{t}^{ij},\,{\rm{if}}\,{y}_{t}=i\\ \,\,\,\,\,\,\,\,\,\,\,\,\,\,\,\,\,\,\,\,\,\,\,\,\,\,\,\,\,\,\,\,\,\,\,\,\,\,\,\,\,\,\,\,\,\,\,\,\,\,{({w}^{ij})}^{T}\phi ({x}_{t})+{b}^{ij}\le -\,1+{\xi }_{t}^{ij},\,{\rm{if}}\,\,{y}_{t}=j\\ \,\,\,\,\,\,\,\,\,\,\,\,\,\,\,\,\,\,\,\,\,\,\,\,\,\,\,\,\,\,\,\,\,\,\,\,\,\,\,\,\,\,\,\,\,\,\,\,\,\,\,{\xi }_{t}^{ij}\ge 0,j=1,\ldots ,l\end{array}$$

where the training data *x*_*i*_ are mapped to a higher dimensional space by the function *ϕ* and *C* is the penalty parameter.

When Eq. 7 is solved, there are decision k functions:

2 $$\begin{array}{c}{({w}^{1})}^{T}\phi (x)+{b}^{1}\\ \vdots \\ {({w}^{k})}^{T}\phi (x)+{b}^{k}\end{array}$$

We find the largest value of the decision function in the class

3 $${\rm{class}}\,{\rm{of}}\,x\equiv \text{arg}{\max }_{i=1,\ldots ,k}({({w}^{i})}^{T}\phi (x)+{b}^{i})$$

Through feature selection, the number of data samples in the experiment is 72 and the number of features is 30, which is consistent with the characteristics of small sample and high dimension. It also indicates that OVR-SVM is very suitable for the three-class classification.

**Figure 2:**
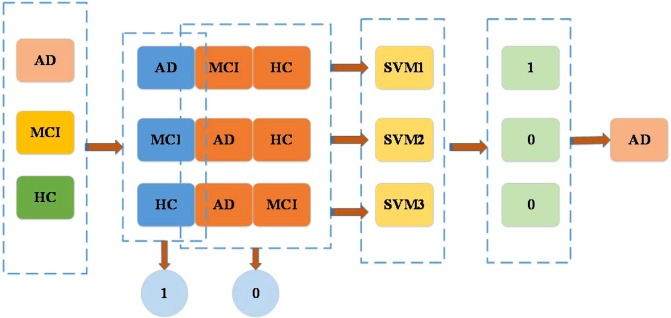
The process of separating AD from HC, MCI, and AD.

In this process, we use three two-class classifiers, the first two-class classifier is HC as the case, MCI and AD as the counterexample, the second two-class classifier is MCI as the case, HC and AD as the counterexample, the third two-class classifier is AD as the case, HC and AD as the counterexample. Figure 2 shows the process to distinguish AD from HC, MCI, and AD.

We also carry out two-two classifications for HC, MCI, and AD by usingthe SVM algorithm.

### Classification and performance metrics

In a pair of training and testing groups, high classification rates may be contingent, so in order to evaluate the prediction performance of the model and reduce over-fitting, we cross-validate the data by 5 folds. The principle of K-fold cross-validation is to divide the whole data into k parts of equal size. Using the model of other k-1 subsets to train classifiers, one of the K parts is tested^12^. In this experiment, the evaluation model uses the following evaluation indicators: Accuracy, Precision, Recall, F1-score. Table 1 lists the confusion matrix of three classification. Each performance is defined in Eqs. 4–7.

4 $${\rm{Accuracy}}=\frac{{T}_{A}+{T}_{B}+{T}_{C}}{T+F}$$

5 $${\text{Recall}}_{classA}=\frac{{T}_{A}}{{T}_{A}+{F}_{AB}+{F}_{AC}}$$

6 $${{\rm{Precision}}}_{{\rm{class}}A}=\frac{{T}_{A}}{{T}_{A}+{F}_{BA}+{F}_{CA}}$$

7 $${F}_{1}-{\rm{score}}=\frac{2\times {\rm{Precision}}\times {\rm{Recall}}}{{\rm{Precision}}+{\rm{Recall}}}$$

**Table 1:** Confusion matrix of three classification.

	Predicted Class A	Predicted Class B	Predicted Class C
Actual Class A	True A (*T*_*A*_)	False A&B (*F*_*AB*_)	False A&C (*F*_*AC*_)
Actual Class B	False B&A (*F*_*BA*_)	True B (*T*_*B*_)	False B&C (*F*_*BC*_)
Actual Class C	False C&A (*F*_*CA*_)	False C&B (*F*_*CB*_)	True C (*T*_*C*_)

## Results

The brain MR imaging data of 72 subjects (mean age:76.3 ± 7.7 years, range: 55.8–95.9 years, meal/female: 40/32) used in this paper are obtained from the Alzheimer’s disease Neuroimaging Initiative (ADNI database (adni.loni.usc.edu), including T1 and T2 structure data, resting state fMRI with eyes open, field map. In the present study, 24 subjects per groups in three classes of HC, MCI and AD were analyzed in this study. Table 2 lists the demographics of all this subjects.

**Table 2:** Basic Information of Sampled Subjects.

Subjects	HC	MCI	AD
Number	24	24	24
Gender(M/F)	16/8	12/12	12/12
Age(mean ± std)	76.3 ± 9.4	76.7 ± 8.7	76 ± 3.8

**Figure 3:**
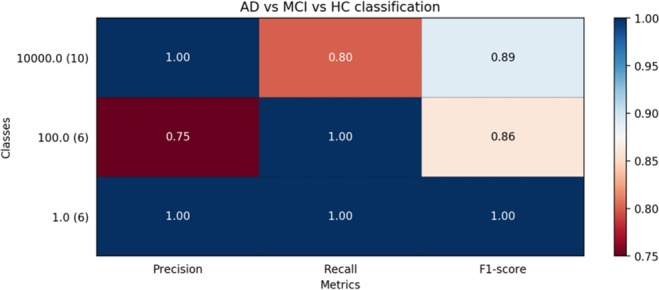
The AD vs. MCI vs. HC classification performance metrics report. Note: 10000.0 stands for AD; 100.0 stands for MCI; 1.0 stands for HC.

In this paper, the final feature vectors, which are obtained after dimension reduction using LR_REF, are classified by SVM. A total of 2,160 feature vectors (72 subjects × 30 features) are used for classification. The state recognition of HC, MCI, AD is performed with the three two-class SVM classifiers. We use the SVM classifier which is implemented by and choose Linear as kernel.The parameters of SVM are determined by 5-fold cross-validation method. The classification results are summarized in Table 3. As can be seen form the Table 3, the OVR-SVM classifier achieved the accuracy of 89% for classification of three groups of HC, MCI, and AD. Moreover, we further applied two typical methods, namely, logistic regression (LR) and K-nearest neighbor (KNN) in Alzheimer’s disease recognition to the same imaging data for a comprehensive comparison. The classification results are summarized in Table 3, which shows that the proposed method achieves better performance than other two methods. The AD vs. MCI vs. HC classification performance metrics are showed in Fig. 3.

**Table 3:** The three-classification average accuracy of different classifier.

Classes	Target	Classifier	Accuracy (%)
Three classes	AD vs. MCI vs. HC	SVM	89.0%
		LR	88.0%
		KNN	71.0%

To estimate the generalization ability of our proposed method, experiments are also performed on three binary classification tasks (HC vs. AD, MCI vs. AD, and HC vs. MCI). The classification accuracies of two classes are 98.0% for AD vs. HC, 92.0% for MCI vs. AD, and 95.5% for HC vs. MCI. Similarly, we further applied logistic regression (LR) and K-nearest neighbor (KNN) to the same imaging data for two-class classification as a comparison. The classification results are summarized in Table 4, which shows that the proposed method achieves better performance than other two methods.

**Table 4:** The two-classification average accuracy of different classifier.

Classes	Target	Classifier	Accuracy(%)
Two classes	AD vs. HC	SVM	**98.0%**
		LR	97.0%
		KNN	92.0%
	MCI vs. AD	SVM	**92.0%**
		LR	92.0%
		KNN	92.0%
	HC vs. MCI	SVM	**95.5%**
		LR	91.0%
		KNN	81.0%

The brain regions corresponding to the 30 features involved in classification. With HCPMMP’s rules for dividing brain regions, the number of the brain region in the right brain is 1–180, and the number of the brain region in the left brain is 181–360. Because the brain is symmetrical, the brain region of the left brain can also be found in the right brain.

**Figure:**
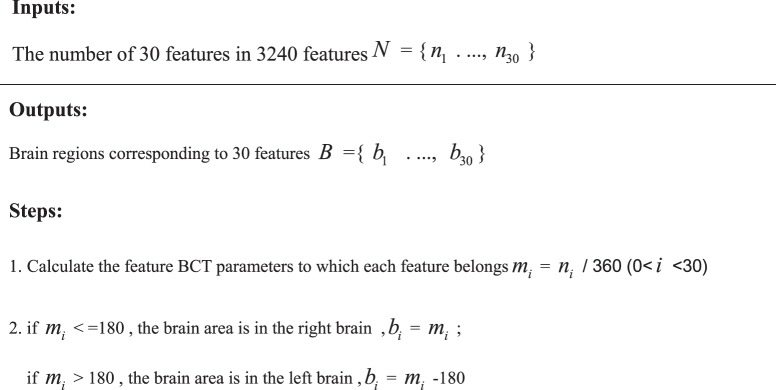
**The step of calculating the brain region.**

In the three-class classification and two-classification of Alzheimer’s disease, we used the 30 features corresponding to the 24 cortical areas in Table 5.

**Table 5:** The information of 30 features corresponding to 24 cortical areas.

Feature	Area ID	Hemisphere	Area
252	72	R	10d
537	177	L	TE1m
792	72	R	10d
851	131	R	TGd
1264	184	L	V2
1278	198	L	FFC
1369	289	L	MI
1391	311	L	TGd
1485	45	R	7Am
1515	75	R	45
1599	159	R	LO3
1603	163	R	VVC
1676	236	L	6 v
1720	280	L	OP4
1788	348	L	lg
1811	11	R	PEF
1893	93	R	OFC
2087	287	L	TA2
2106	306	L	PHA1
2232	72	R	10d
2320	160	R	VMV2
2329	169	R	FOP5
2462	302	L	PeEc
2502	342	L	31a
2613	93	R	OFC
2655	135	R	TF
2718	198	L	FFC
2722	202	L	PIT
2789	269	L	A10p
2822	302	L	PeEc

**Figure 4:**
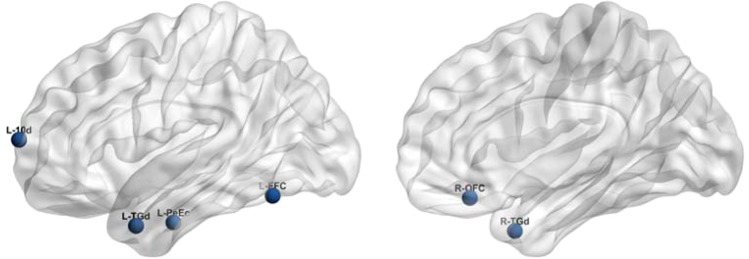
The five core cortical areas’ specific distribution in the brain.

As shown in Table 5, we further analyzed the information of 30 features and then found the five key cortical areas, and each and each cortex area corresponded to two or more features, namely Fusiform Face Complex (L-FFC), Area 10d (L-10d), Orbital Frontal Complex (R-OFC), Perirhinal Ectorhinal (L-PeEc) and Area TG dorsal (L-TGd,R-TGd). The corresponding characteristics of specific key areas are shown in Table 6. Their specific distribution in the brain is shown in Fig. 4.

**Table 6:** The information of 11 features corresponding to the 5 cortical areas.

Area Name	Parcel Index	Feature	Area Description	Other Name
FFC	18	1278, 2718	Fusiform Face Complex	FFA, FG2
10d	72	252, 792, 2232	Area 10d	10, Fp1, Fp2
OFC	93	1893, 2613	Orbital Frontal Complex	11 m, 13b, 13 m, 14r, Fo1
PeEC	122	2462, 2822	Perirhinal Ectorhinal	ATFP, AFP1, 35,36
TGd	131	851, 1391	Area TG dorsal	TG

In order to further analyze the five core Cortical areas, the 11 features corresponding to the five Cortical areas of FFC, 10d, OFC, PeEc and TGd are selected from 30 features corresponding to the 24 Cortical areas, which are used to classify HC, MCI, and AD. Subsequently, we use the 5-fold cross-validation of SVM and LR to classify these separately. From Table 7, the accuracies of the classification in SVM and LR with 11 features are 80% and 78%, respectively.

**Table 7:** The classification accuracies corresponding to different brain areas and features.

Modle	Set 1	Set 2	Set 3
SVM	89%	80%	48%
LR	88%	78%	49.8%

Note:

Set 1: classification accuracies in SVM and LR with 24 brain areas and 30 features;

Set 2: classification accuracies in SVM and LR with 5 core brain areas and 11 features;

Set 3: classification accuracies in SVM and LR with 11 features and random 5 brain areas from 24 brain areas except 5 core brain areas.

In addition, the accuracies of the classification in SVM and LR with 30 features of 24 cortical areas are 89% and 88%, respectively. Furthermore, in order to analyze the role of the features of five cortical areas in classification, we randomly select the corresponding features of the five cortical areas in the remaining 19 cortical areas to calculate the accuracy of classification. The training and test are repeated 10 times to get the average accuracies for SVM and LR. The classification results of Accuracy_3 were given in Table 7.

From Table 7, the classification accuracies of Set 2 are closer to that of Set 1, but the classification accuracies of Set 3 are much lower than that of Set 1. Obviously, when the features are taken from five Cortical areas of FFC, 10d, OFC, PeEc and TGd, the classification accuracy is high than random five cortical areas. Therefore, we observe that the five cortical areas have a great impact on the results of the three-class classification.

## Discussion

Most previous studies focused on the two-class classification between HC, MCI, and AD, and they have achieved great accuracy. With the imaging data of ADNI database, some studies also reported recognition results of three-class classification between HC, MCI, and AD. As shown in Table 8, our method obtained higher accuracy than previous studies using old brain parcellation methods. It shows that our parcellation scheme benefits the classification of HC, MCI, and AD. It also proves that the division of brain regions of JHCPMMP is more scientific and effective than other methods.

**Table 8:** Comparison of classification accuracy for recent studies.

classes	Authors	Target	Modality	Machine Learning	Brain Segmentation Method	Accuracy
Two classes	Suk *et al*.^18^	AD vs. HC	MRI + PET	Multi-Kernel SVM	93 regions	95.9%
		MCI vs. HC				85.0%
		MCI-C vs. MCI-NC				75.8%
	Ortiz *et al*.^19^	AD vs. HC	FDG-PET + sMRI	SVM (Linear)	42 subcortical regions	92%
		MCI vs. AD				84%
		HC vs. MCI				86%
	Li *et al*.^20^	AD vs. HC	MRI + PET	RBM and SVM	93 volumetric regions	91.4%
		MCI vs. HC				77.4%
		AD vs. MCI				70.1%
		MCI.C vs. MCI.NC				57.4%
	Khedher *et al*.^21^	HC vs. AD	sMRI(T1)	SVM(Linear)	SPM8	87.12%
		HC vs. MCI				77.62%
		MCI vs. AD				85.41%
	Our Method	AD vs. HC	fMRI	Linear-SVM	J-HCPMMP	**98.0%**
		MCI vs. AD				**92.0%**
		HC vs. MCI				**95.5%**
Three classes	Quintana *et al*.^22^	MCI vs. AD vs. HC	NPR	ANN	55 regions	66.67%
	Zhang *et al*.^23^	MCI vs. AD vs. HC	MRI	SVM (RBF)	66 volumetric features	81.5%
	Tong *et al*.^24^	MCI vs. AD vs. HC	sMRI(T1) + PDG-PET + CSF + Genetics	NGF + SVM	83 anatomical regions	60.26%
	Lama *et al*.^25^	MCI vs. AD vs. HC	sMRI(T1)	PCA + RELM	FreeSurfer 5.3.0	61.58%
	Son *et al*.^26^	MCI vs. AD vs. HC	sMRI(T1) + rs-fMRI	Random Forest	10 subcortical regions	53.3%
	Our Method	MCI vs. AD vs. HC	fMRI	Linear-SVM	J-HCPMMP	**88.0%**

As shown in Table 7, when the features are taken from five cortical areas of FFC, 10d, OFC, PeEc and TGd, the classification accuracy is high than that using five random cortical areas. Therefore, the five cortical areas have a great impact on the results of the three-class classification. And this finding has been confirmed in previous clinical papers.

Zebrowitz^13^ observed lower activation, specificity, and resting blood flow for older adults than younger adults in the fusiform face area (FFA) but not in other regions of interest, and then the facial selection mechanism of the elderly was uncoordinated. Bludau *et al*.^14^ found that Fp1 and Fp2 have different contributions to functional networks. Fp1 was involved in cognition, working memory and perception, whereas Fp2 was part of brain networks underlying affective processing and social cognition. Grabenhorst *et al*.^15^ pointed out that OFC can affect people’s function of feeling happiness, pain, and reward and punishment. Ding *et al*.^16^ found that human TPC actually includes anterior parts of areas 35, 36, and TPC seems to be involved in social and emotional processing to a large extent, including facial processing, recognition and semantic memory. Olson *et al*.^17^ studied that TGd may combine complex and highly processed perceptual input with visceral emotional response. Thus, there five areas all have been confirmed to be involved in human facial processing, emotional perception and memory function. Therefore, our results were in line with those reported in previous studies, showing significant importance to further explore the treatment strategies of Alzheimer’s disease, and carry out early intervention to delay the deterioration of the disease.

## Conclusion

We propose a method JMMP-LRR which combines LR-RFE and JHCPMMP for three classifications of AD patients. fMRI data is processed by JHCPMMP to obtain small samples, ultra-high-dimensional data, these data directly involved in classification will cause too long running time and low classification accuracy, JMMP-LRR can solve the problem very well. The features obtained by using LR-RFE as feature extraction were more recognizable for the three classifications of AD patients, and could achieve high classification accuracy. By analyzing the features obtained by LR-RFE, we find 5 brain regions were sensitive to AD patient identification: L-FFC, L-10d, R-OFC, L-PeEc, (L-TGd, R-TG). Only use the functional features of these 5 brain regions, we could achieve high accuracy. The accuracies of the two experiments using the JMMP-LRR method were higher than the current method. It also proves that JHCPMMP is better than other brain partitioning methods in identifying patients with AD.

## Data Availability

Data collection and sharing for this project is funded by the Alzheimer’s Disease Neuroimaging Initiative (ADNI) (National Institutes of Health, USA).
